# Synchrotron microtomography applied to the volumetric analysis of internal structures of *Thoropa miliaris* tadpoles

**DOI:** 10.1038/s41598-020-75993-8

**Published:** 2020-11-03

**Authors:** G. Fidalgo, K. Paiva, G. Mendes, R. Barcellos, G. Colaço, G. Sena, A. Pickler, C. L. Mota, G. Tromba, L. P. Nogueira, D. Braz, H. R. Silva, M. V. Colaço, R. C. Barroso

**Affiliations:** 1 Laboratory of Applied Physics to Biomedical Science, State University of Rio de Janeiro, Rio de Janeiro, Brazil; 2 Laboratory of Herpetology, Federal Rural University of Rio de Janeiro, Rio de Janeiro, Brazil; 3 Elettra/Sincrotrone Trieste S.C.P.a., Trieste, Italy; 4 Oral Research Laboratory, Institute of Clinical Dentistry, University of Oslo, Oslo, Norway; 5 Nuclear Engineering Program/COPPE, Federal University of Rio de Janeiro, Rio de Janeiro, Brazil

**Keywords:** Imaging techniques, Data acquisition, Data processing, Image processing, Software

## Abstract

Amphibians are models for studying applied ecological issues such as habitat loss, pollution, disease, and global climate change due to their sensitivity and vulnerability to changes in the environment. Developmental series of amphibians are informative about their biology, and X-ray based 3D reconstruction holds promise for quantifying morphological changes during growth—some with a direct impact on the possibility of an experimental investigation on several of the ecological topics listed above. However, 3D resolution and discrimination of their soft tissues have been difficult with traditional X-ray computed tomography, without time-consuming contrast staining. Tomographic data were initially performed (pre-processing and reconstruction) using the open-source software tool SYRMEP Tomo Project. Data processing and analysis of the reconstructed tomography volumes were conducted using the segmentation semi-automatic settings of the software Avizo Fire 8, which provide information about each investigated tissues, organs or bone elements. Hence, volumetric analyses were carried out to quantify the development of structures in different tadpole developmental stages. Our work shows that synchrotron X-ray microtomography using phase-contrast mode resolves the edges of the internal tissues (as well as overall tadpole morphology), facilitating the segmentation of the investigated tissues. Reconstruction algorithms and segmentation software played an important role in the qualitative and quantitative analysis of each target structure of the *Thoropa miliaris* tadpole at different stages of development, providing information on volume, shape and length. The use of the synchrotron X-ray microtomography setup of the SYRMEP beamline of Elettra Synchrotron, in phase-contrast mode, allows access to volumetric data for bone formation, eye development, nervous system and notochordal changes during the development (ontogeny) of tadpoles of a cycloramphid frog *Thoropa miliaris*. As key elements in the normal development of these and any other frog tadpole, the application of such a comparative ontogenetic study, may hold interest to researchers in experimental and environmental disciplines.

## Introduction

Since the introduction of microtomography (micro-CT) as a tool for anatomical investigations of vertebrates, several studies have used the reconstructed images as a way to present morphological information and to perform comparative studies based on them^1–4^. Recent studies have focused on adult morphology and on descriptive aspects of biology of several species of living fishes^5–7^, salamanders^8–10^, caecilians^11–14^, frogs and toads^15–19^, lizards^20–22^, snakes^23–25^, amphisbaenians^26–28^, turtles^29–31^, crocodiles^32–34^, birds^35–37^, and mammals^38–40^, besides fossils of several vertebrate groups^41,42^. On herpetological research, studies that encompass developmental series, together with other purely anatomical studies, represents the most of works reported this way, mainly for anurans and squamates species^43^. However, in developmental studies, is still hard to obtaining complete (or almost) developmental series and/or to discriminate soft tissues (e.g. cartilages, muscles, nervous system and visceral organs) using X-ray based technology^44,45^. Even for anuran amphibians, which have abundant information and material available for tadpoles^46^, and series of development, tomographic studies have been less applied to larval stages than for adults^47^.

Most studies of anuran development are based on whole preparations and cleared and bone-cartilage double-stained specimens^48–50^. These studies, about bones and cartilaginous elements, have become standards for comparing sequence of skeleton development and in systematic surveys^46^. However, they are destructive^51^, because the specimen’s other information (e.g. internal organs and muscles) is lost during the process of preparation. Therefore, if changes of a particular element were to be followed, and one wants, for instance, to follow the amount of bone deposition and ossification, and elaborate area reconstructions have been devised to account for that^52^. One of the advantages of microtomography reconstruction, and software developed for it (as for example, the software used herein; see below), is that volume and other sort of measurements can be performed^43^ to selected tissues and organs.

Different applications of micro-CT and its development have been published previously^53–55^. When using an X-ray microtomograph, factors such as the thickness and the atomic number of the sample determine the level of contrast in the radiographic projections, due to differences in the absorption of the X-ray when passing through the sample. One of the main disadvantages of conventional X-ray imaging techniques for application in biological samples is that these samples generally have low density, causing the emission of hard X-rays from conventional microtomographs to have even less absorption.

In the 1990s, the phase-contrast imaging technique emerged, becoming an effective approach applied to synchrotron micro-CT, capable of providing a better contrast level in radiographic projections^56,57^. Currently, the phase-contrast technique has been widely used in the scientific community, as it is a technique associated with radiography and microscopy and that enhances contrast in soft tissues, specifically at the edges, showing details that could not be seen by the absorption technique^57^.

When radiation encounters the surface and internal structures of a sample, there is an apparent deviation in the propagation of X-rays, caused by the refraction index of radiation in each structure, where this index has an imaginary component (referring to the absorption of X-rays) and a real component (referring to the phase-contrast). For the energy range of hard X-rays, the variations in the phase-contrast component of a low-density material are considerably greater than the variations in the absorption component. Therefore, phase-contrast variation results in an improvement in the contrast through the edges of the structures for low absorption materials, in this case, biological samples^58–60^.

There are several methods to measure X-ray phase-contrast imaging (X-PCI). The physical and technical principles of X-PCI are explained in a number of review articles^61–63^. The propagation-based phase-contrast imaging (PBI) uses free-space propagation (FSP) to encode the phase signal into measured intensities; it is dependent upon both the sample-detector distance and the detector resolution^57,64,65^. If the propagation distance is chosen properly, phase-contrast will result in edge enhancement. Recent developments of X-ray sources in synchrotron laboratories enable them to reach unprecedented characteristics of brilliance and intensity, allowing the application of propagation-based phase-contrast imaging (PBI)^66–68^.

**Figure 1:**
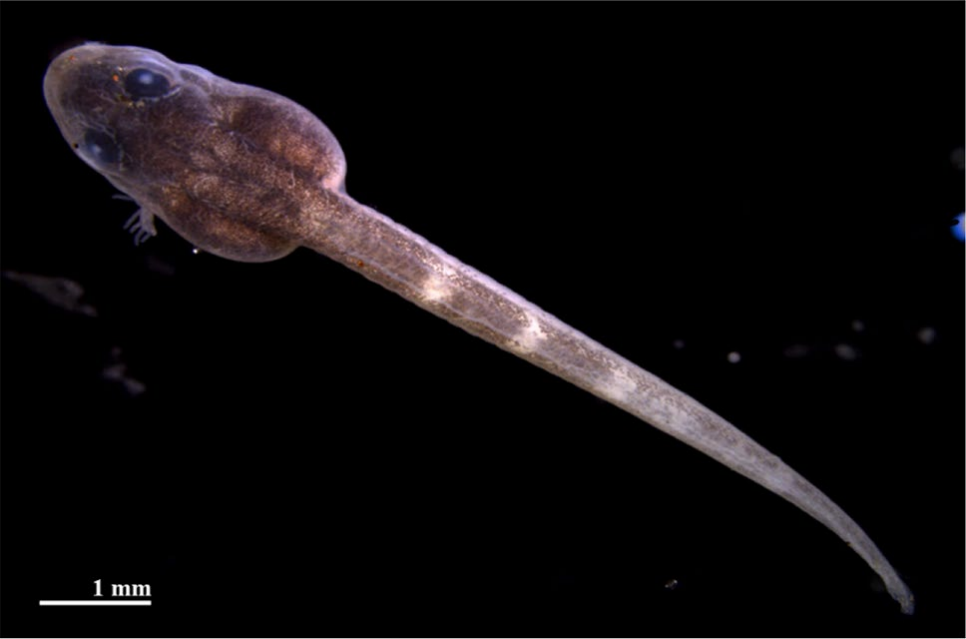
Post-hatching stage of the *Thoropa miliaris* egg seen by optical microscope.

In this study, we show how the use of the synchrotron X-ray micro-CT setup of the SYRMEP beamline of Elettra Synchrotron, in phase-contrast mode, allows reconstruction volumetric data for bone formation, eye development, nervous system and nothocordal changes during the development of tadpoles of a cycloramphid frog, *Thoropa miliaris* (Fig. 1) based on formaldehyde preserved specimens. Our goal is to investigate the ability of phase-contrast micro-CT for visualization of soft tissues and rigid internal structures (like bones) at the same time, rather than to describe the anatomy in detail.

## Methods

### Sample preparation

In this work, specimens of *Thoropa miliaris* were used for this investigation. The samples are part of the natural history collection of the Biology Institute of the Federal Rural University of Rio de Janeiro, and were returned to the collection after the experimental procedure by tomographic scan.

These samples were collected in the municipality of Mangaratiba (Rio de Janeiro, Brazil) under license SISBIO/IBAMA number 10689-1 and euthanized with MS222 (tricaine methanesulfonate), following the recommendations of amphibians euthanasia of the Brazilian National Council for the Control of Animal Experimentation (CONCEA), page 42^69^. No euthanasic process was carried out for this work, as we only used samples preserved in formalin from the university's collection.

The sample included specimens on nine distinct larval and metamorphic stages of development (28, 30, 32, 34, 37, 38, 40, 43 and 44), staged following a table based on external morphology developed by Gosner^70^. Because the tadpoles are stored in formaldehyde 10%, in order to facilitate handling and avoid hazardous inhalation, specimens were previously washed in distilled water and passed in progressive 10 min baths of 20%, 50%, 70%, and absolute ethanol, in which they were stored for the tomography. At the end of this process, the samples were packed in polypropylene tips containing absolute ethanol and under light pressure of a plunger. This protocol of tomographic scanning was developed by us earlier in another study^71^ that also used synchrotron radiation.

### SYRMEP beamline setup and data acquisition

The X-ray phase-contrast CT scans were performed at the SYRMEP beamline of the Elettra Synchrotron^72^, Trieste, Italy. All samples were scanned using the 2 GeV storage ring mode and a white (polychromatic) X-ray beam with the mean energy 25 keV, in combination with a sample-detector distance of 10 cm and exposure time per projection of 100 ms. The X-ray spectrum of the beam was filtered with 0.025 mm of molybdenum to reduce the contribution of low energy X-rays.

Projections were recorded with a 16-bit, air-cooled sCMOS camera (Hamamatsu C11440-22C ORCA-Flash 4.0 v2) (see^73–76^). A 2.2 μm effective pixel size was set, using the variable optical zoom of the detecting system, corresponding to a field of view (FOV) of about 4.5 mm × 4.5 mm.

Using polychromatic radiation allows higher photon flux and thus decreases the scan duration of micro-CT experiments. For each sample in stages 28 to 42, a scan containing 900 projections was collected with a 0.2° angular step over 180° and total scanning time of around 2 min. Larger samples (stages 43 and 44) were scanned off-center over 360° (1800 projections) which allowed almost doubling of the FOV resulting in total scanning time of around 4 min.

### Data processing and analysis

Obtaining high-contrast tomographic data is the first step in 3D analysis. CT data pre-processing and reconstruction were performed using the open-source software tool Syrmep Tomo Project (STP)^77,78^, custom-developed at the SYRMEP beamline tuned up for phase-contrast computed tomography experiments. Before reconstruction of 2D axial slices, the collected projections were pre-processed using dynamic flat fielding correction and ring artifact compensation. Paganin’s single-distance phase-retrieval approach^79^ based on the transport of intensity equation (TIE) combined with the Filtered Back-Projection algorithm and implemented within the STP software has been applied to obtain high quality 3D images, where the different grey-levels are proportional to the electron density of the different tissues inside the sample. Phase-retrieval is a technique for extracting quantitative phase information from X‑ray propagation-based, phase-contrast tomographic images^66^.

**Figure 2:**
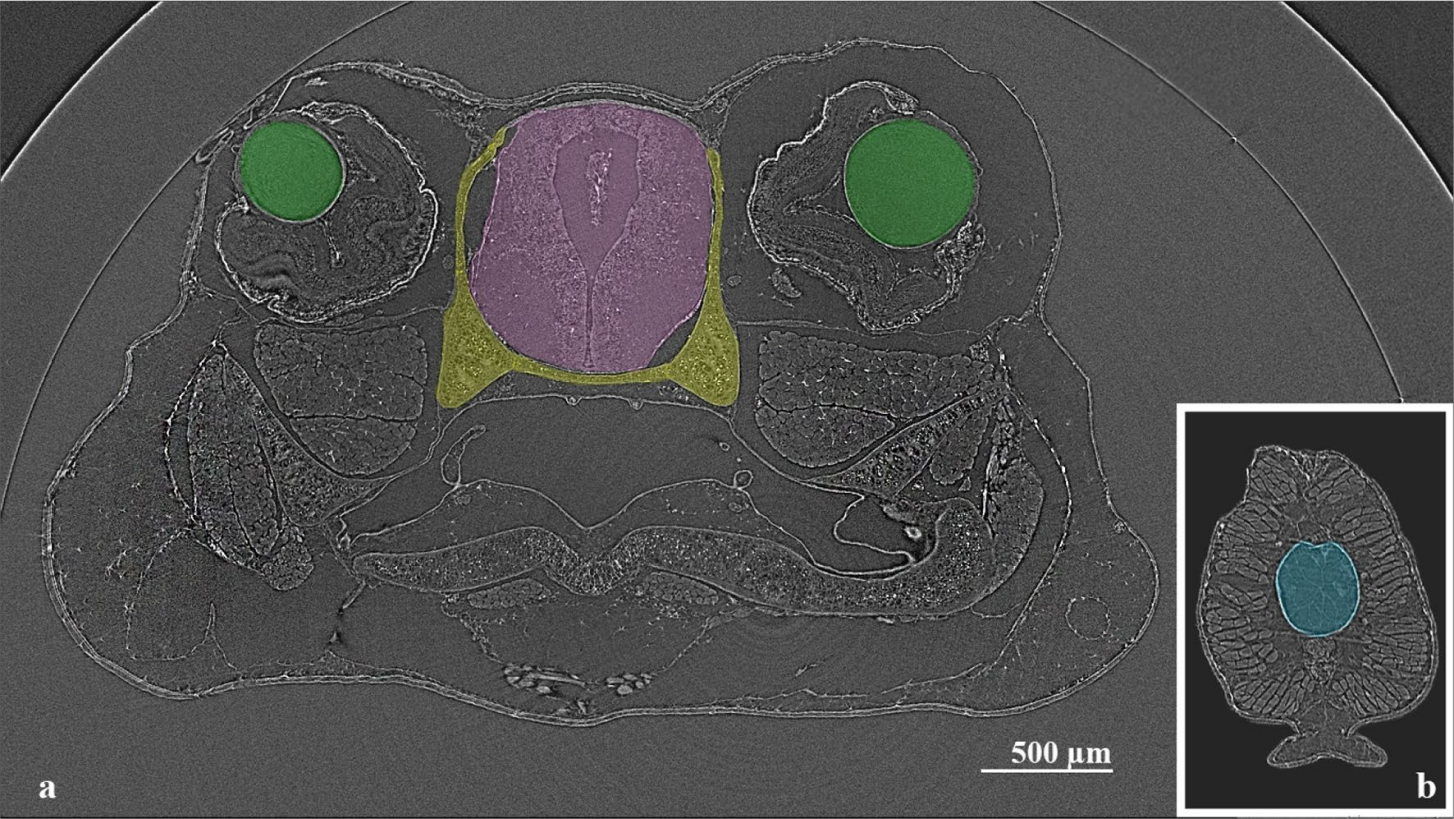
Micro-CT slices of *Thoropa miliaris* tadpole specimen in stage 40: (**a**) Head region slice where the green ROI represents the lens, pink ROI the central nervous system (cranial region) and, yellow ROI the endolymphatic calcifications/skeletal tissue. (**b**) Caudal region slice where the blue ROI represents the notochord.

**Figure 3:**
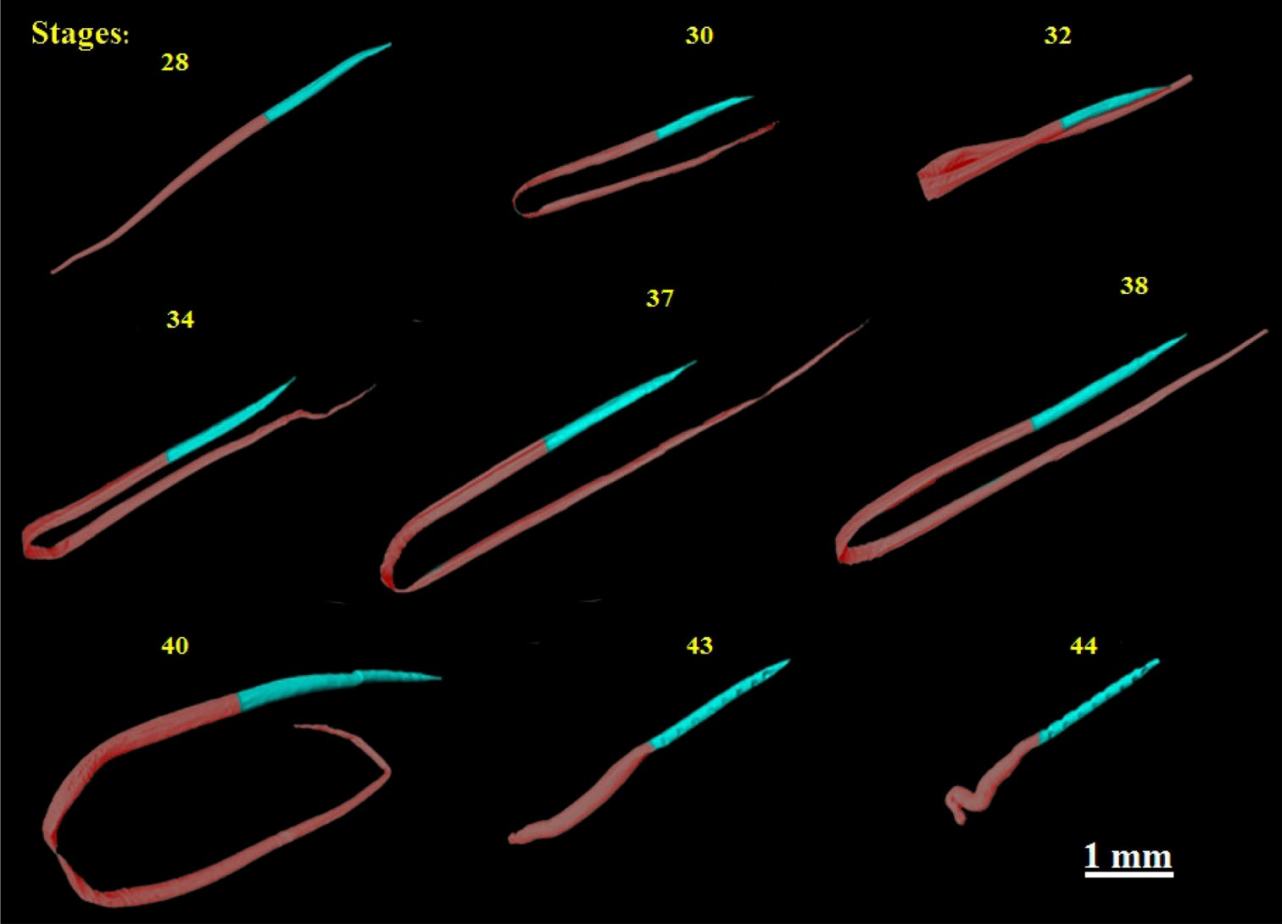
Notochord in different stages of development (28 to 44) in both anterior and posterior regions of the body: blue identifies the thoracic region and red the caudal region. The vent tube is located at the division between two regions.

**Figure 4:**
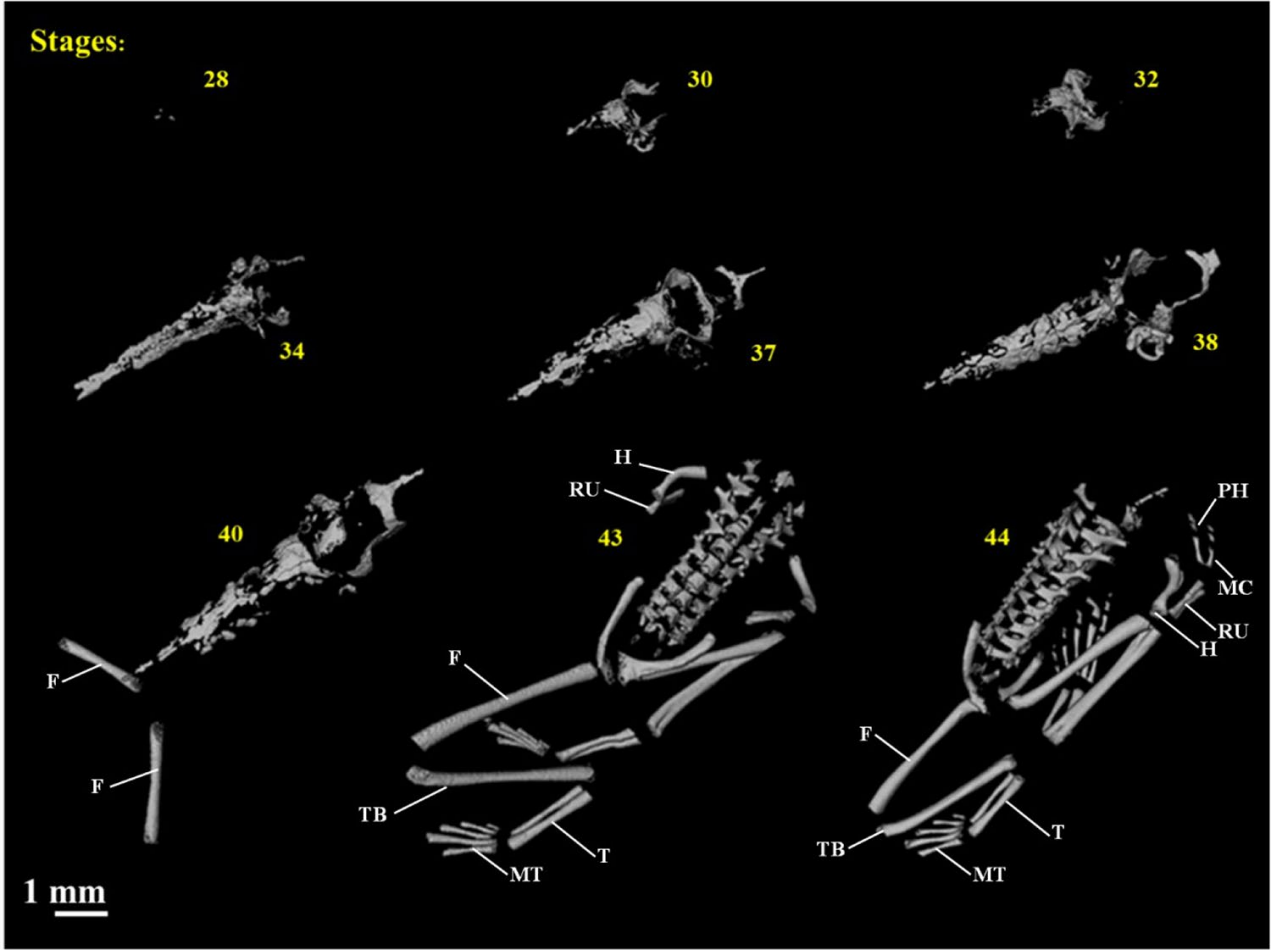
3D segmentation of ECD and bone tissue showing the development of the amphibian skeleton, showing fêmur (F), humerus (H), metacarpus (MC), metatarsus (MT), phalanges (PH), radio-ulna (RU), tarsos (T) and tibiofibular (TF) in the most advanced stages.

**Figure 5:**
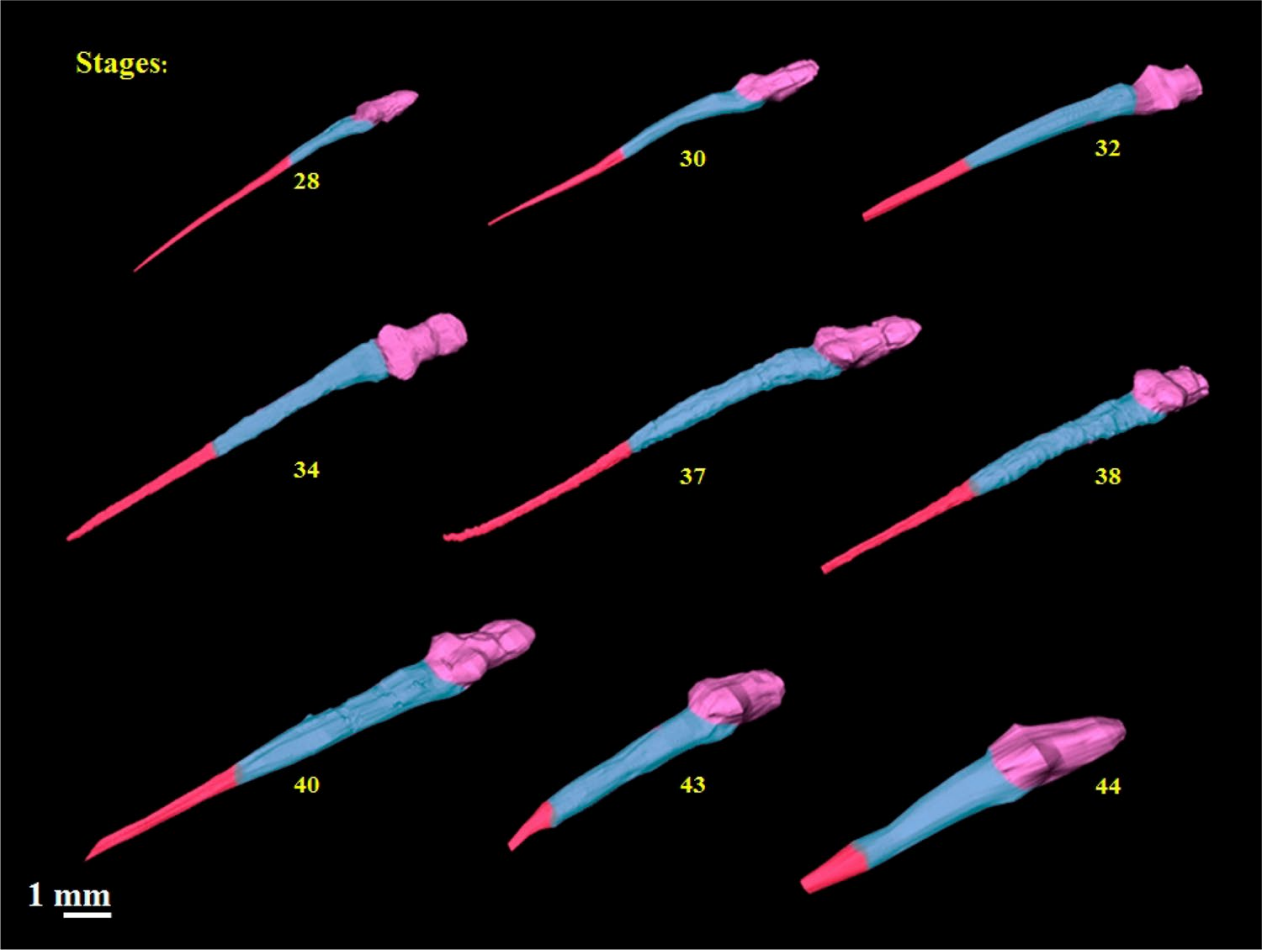
*Thoropa miliaris *segmentation of the 3D geometry of central nervous system of each stage analyzed. Note the large variation of length of the CNS during the development of tadpole, passing metamorphic climax (between 38 and 41) until complete regression of the tail. Pink represents the cranial region, blue the thoracic region, and red the caudal region.

**Figure 6:**
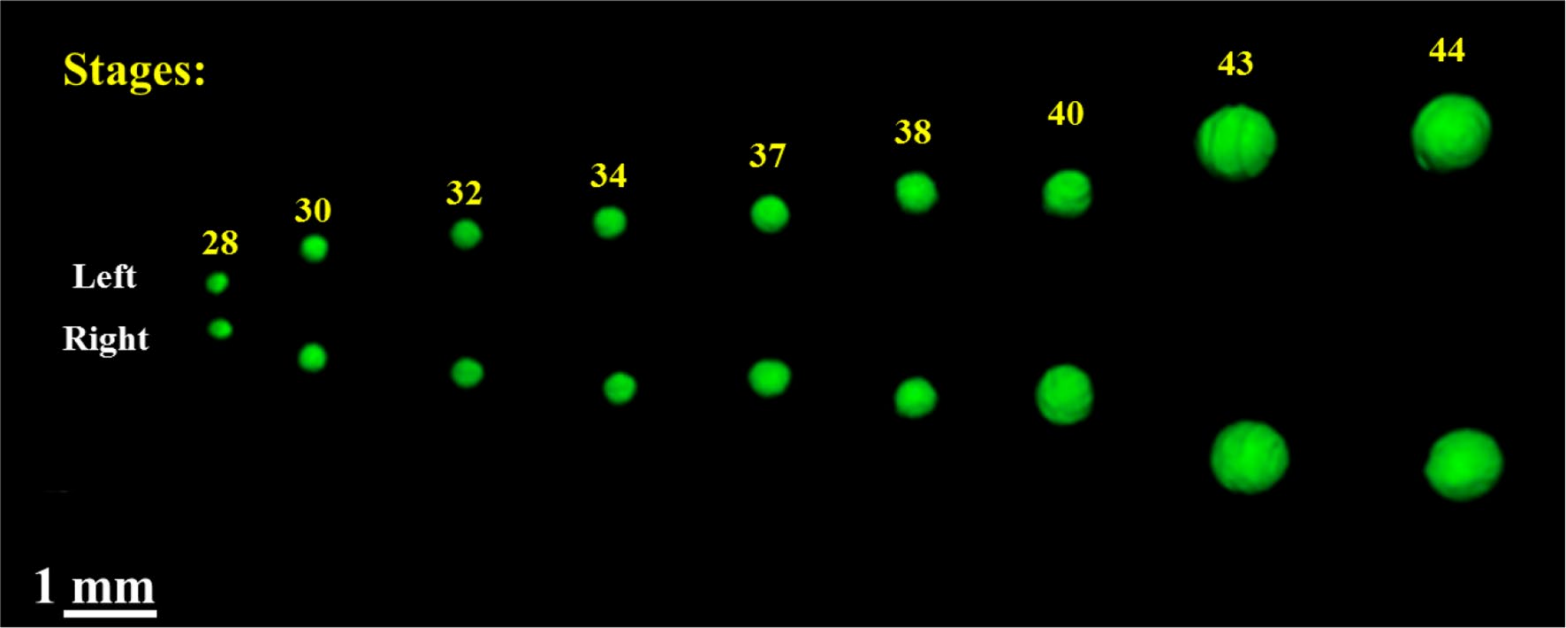
Lens left and right eye at each stage showing its progressive development.

Another important part is the data processing and analysis of the reconstructed tomographic volumes. Post-reconstruction data treatment requires segmentation of the investigated tissues, organs, or bone elements by applying the appropriate algorithms for distinguishing them in the regions of interest (ROI) (Fig. 2) and then its volume of interest (VOI), defined as an ROI stack. After this procedure, the different tissues were segmented semi-automatically with the software Avizo Fire 8^80^ and was realized the quantitative analysis of each region. Results are presented in Figs. 3, 4, 5, 6.

## Results

### Notochord

The notochord shares many cellular properties with cartilage; however, while the latter does not show up in micro-CT analyses of cleared and stained specimens, the former does, allowing its segmentation and as volumetric quantification. Our images show the notochord folded because it was bent, in order to fit larger specimens into the tube so it could be detected by the tomographic projections in the field of view of the sCMOS camera. The images clearly show the elongation of the notochord as the tadpole grows. Growth is mainly observed towards the tail, and that the organ gets relatively longer as stages progress. In the latter stage (stage 38) the notochord reaches its longest size. From this stage on, as the whole tail is reabsorbed (via apoptosis), the notochord also decreases in size. The image below (Fig. 3) shows the notochord in different *Thoropa miliaris* tadpoles stages, marking the ventral tube as a divisor between the cranial and caudal region of notochord.

### Calcification, bone tissue, and skeletal development

The skeleton of *Thoropa miliaris*, as that of most anurans, is made up of cartilaginous tissue during most of the early development. The use of staining methods for cartilaginous tissue visualization in X-rays techniques is common^51^. However, as calcium carbonate is deposited in the process of dermal and cartilaginous bone formation, the technique here employed allowed us to detect this process and its progress along the stages without staining methods. Here we report only part of our results. Because of its biological implication, the correlation between the Endolymphatic Calcium Deposit (ECD, that stores calcium in an acellular form) and ossification will be explored further elsewhere.

At stage 28 a small amount of calcium was already detectable near the ECD closer to the skull’s occipital region (Fig. 4). As development progresses, more calcium carbonate is accumulated in the ECD and bone replaces cartilage in the formation of the vertebral column, chondrocranium, and limbs. In latter stages (43 and 44), the hind- and forelimbs are well developed. However, at stage 40 the amount of bone measured decreased, as compared to the amount detected at stages 43 and 44. This seems to indicate a limitation in detecting small amounts of ossification in the phalanges, that may already be present, but do not show in the reconstruction as seen in Fig. 4.

### Central nervous system

The brain and spinal cord are part of the central nervous system which consists of a complex network of nerve cells and tissue that enables an organism to interact with its surroundings by perceiving reacting to it (behaving). When tadpole are compared, it is noticeable that the length and volume of the brain and spinal cord increases in size and volume (see Fig. 5) first in low increments, and later sharp ones. Following apoptosis of the tail (marking the onset of metamorphosis), the size of the spinal cord decreases. Nevertheless, the size and the volume of the brain and the remaining spinal cord increases (Fig. 5).

### Lens

Throughout the development of *Thoropa miliaris* tadpole the volume of its lens (Fig. 6) increased, at the same time its shape changed from nearly spherical to an ellipsoid with low eccentricity.

## Discussion

### Phase-contrast and the study of development in anurans

The effects of the phase-contrast applied to the samples are visible on the scans, allowing the edges of the internal organs to be more evident. With the use of this technique it was possible to make detailed observations of several structures. With the phase-contrast technique in the process of three-dimensional segmentation of several internal structures without the use of chemical stains by contrast agents, reducing sample preparation time.

The phase-retrieval algorithm was fundamental for visualization and tomographic rendering. Phase effects are intensely present and can be noticed in the significant edge effects, caused by the difference in refractive indices of distinct tissues. The 3D segmentation performed by the software Avizo allowed the visualization and quantification of the volume of different structures and their measurement, the implications of which are discussed below.

**Figure 7:**
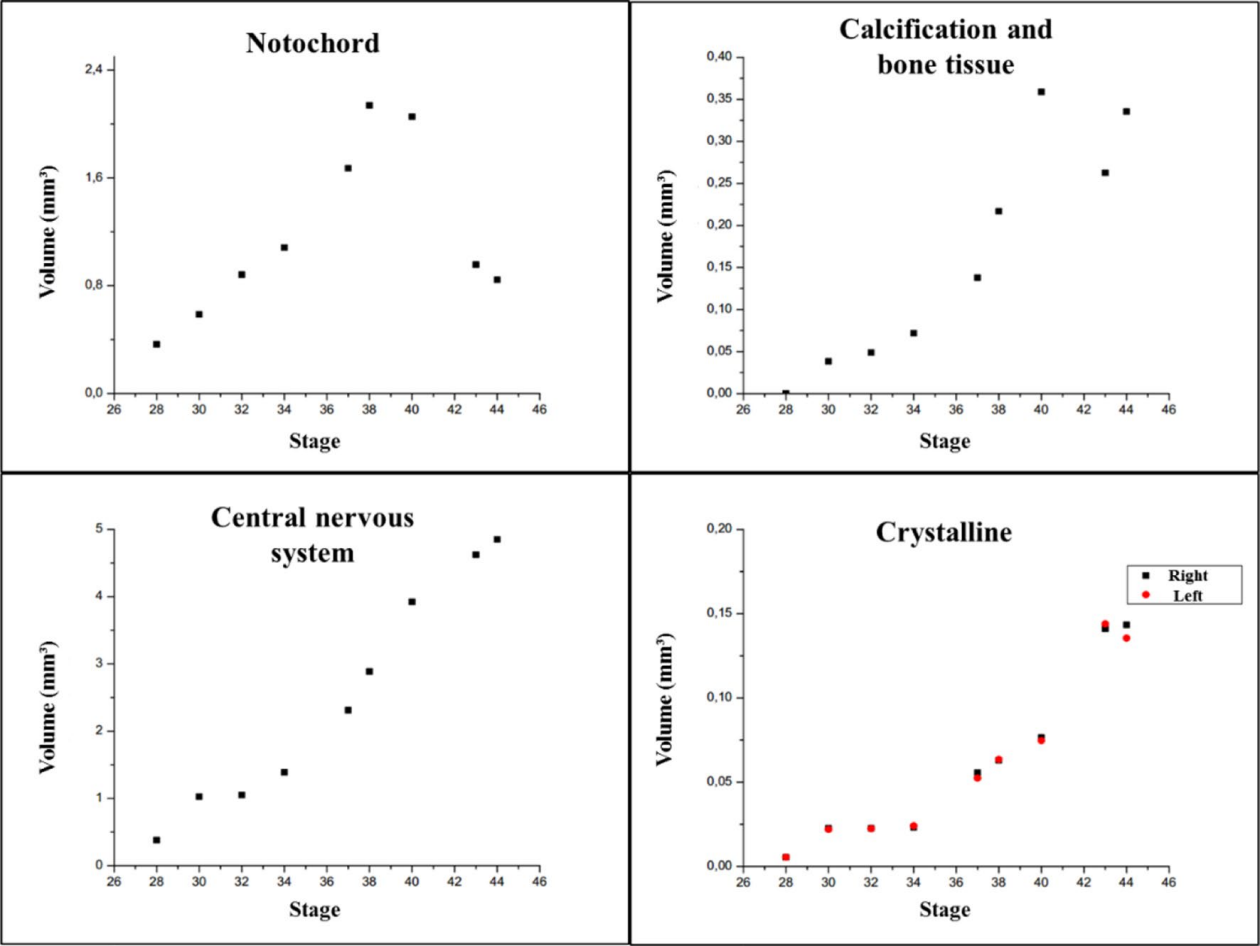
Estimated volume by Avizo software in each analyzed region.

For each sample, three tomographic acquisitions (head, body and tail) were required, because samples were large size compared to the detector screen of the SYRMEP line, which was 4 × 4 mm. After the radiographic acquisitions, were realized the reconstruction of these images and the slices sequence (reconstructed images) of each part of the tadpole’s body were merged using the Avizo software, being a file with the size around 300 GB. For such processing, a high-performance computer and processing hours are required. After this process, we have a body-whole micro-CT of the specimen and analyze what was needed. The image below (Fig. 7) shows the estimated volumes by Avizo software in analyzed regions of *Thoropa miliaris* tadpole, after this computational process.

### Notochord

The notochord is an embryonic structure (in most cases) unique to the Phylum Chordata^81,82^. Within this group, among craniate vertebrates, by the end of the embryonic period of all the species of Osteichthyes, the notochord is replaced by endochondral bone associated with the formation of the vertebral column, and it is not present in most adults specimens^82–84^. Besides having a supporting role, the notochord also functions as signaling organ, responsible for controlling the differentiation and early development of several other tissues, from muscles to the pancreas (see^82,85,86^ for a review). Although notochord resembles cartilaginous structures, at the cellular level these tissues present some structural differences^86^, such as the retention of large vacuoles filled with hydrated material in notochordal cells^84,85^. Possibly because of these differences, amphibian notochord cells are more difficult to have efficient staining methods. However, visualization of the notochord in phase-contrast micro-CT scans is simple and does not require chemical staining. The location of the notochord in the animal's body also aids in visualize this structure in the tomographic slices, due to the difference in density of notochord with neighboring tissues.

Although the fate of the notochord is fairly well known^82^, visualization of the whole processes involved and its development usually requires a complex set of histological techniques and early histology based tomographic reconstruction^87^. Our study shows that its possible a good quality visualization of the notochord along development of frogs and that along the different stages, it grows and that its anterior region thickens. In later stages, its posterior end is resorbed which can be easily documented. Moreover, we were able to document that its internal apoptosis may happen earlier and as a result, the tissue that envelops it at the tail’s end gets squished and folded (see Fig. 3 stages 43 and 44). With a larger sample size, it will be possible to quantify the amount of change data takes place in the notochord during development.

### Calcification, bone tissue and skeletal development

As the larval development of the anurans progress, bone tissue appears either in the form of dermal bone (e.g. squamosals, nasals, frontoparietals, and maxillaries), or as perichondral (long bones, e.g. the femur) or endochondral (vertebra, sphenethmoid) ossification (e.g.^52^). When measuring volumetric changes to evaluate the calcium content of bones, this distinction is fundamental. In the present case, for instance, we were more concerned with the content of the acellular ECD, that is solid, and many regions of perichondral and endochondral bones. Unless in the process of rendering the images as single X-rays one can average the proportion of perichondral bone deposits is taking place, the quantification will be imprecise. In the case of the vertebrae, however, which develop via an endochondral process, the degree of precision is greater. Another concern with the use of this technique for accessing volumetric data in small organisms is the ability to render really small bones, like the tiny phalanges, that do not appear in the images. This problem may be less important when dealing with CT-scan reconstruction in larger vertebrates.

### Central nervous system

Because of the dual life history of most frogs, with aquatic larvae and terrestrial adults, via metamorphosis, the nervous system also suffers dramatic reconfigurations and changes. Some of such obvious changes related to apoptosis in distinct regions, like the tail and larval mouthparts, including muscles that disappear. Another group of changes includes the development of new organs, like limbs and auditory apparatus that appear towards the end of metamorphosis. In tandem with these changes, it is expected that the accompanying nervous system also changes. So, in order to control the developing limbs, as an example, a complex of brachial and pelvic plexus develop, at the same time that the nerves controlling the tail (and some other muscles) are reabsorbed. It is important to point out that, abdominal, and several other muscles also appear at metamorphosis, all demanding nerve, and central nervous system (CNS) control. Although we did not measure volume for nerves, especially the smallest ones, the images in Fig. 5 allow inferring part of these changes taking place at the caudal region, which over time shrinks and disappears, while the rest of the CNS increases in relative volume and thickness in the 3D image visualizations.

### Lens

Changes in the eyes of anurans during development, mainly relating to the transition from water to terrestrial habitat is well documented^88^, the changes that take place in the lens during metamorphosis is far less studied^89^. This may result from difficulties relating to the histological techniques associated with fixing, staining and sliding the organ for examination of its parts^90^, mainly because of the hardness of the crystalline proteins of the lens. We only found three works that dealt with changes during the eye development^89,91,92^. Only the work of Sivak and Warburg^89^ dealt with changes in the lens.

As tadpoles grow the lens also grows and changes its shape, from almost spherical to ellipsoid. As a result, the adult morphology is more suited to focus images in the air, perceive predators, and, perhaps most importantly, adjust focus for preying on moving animals.

## Conclusions

This work shows the *Thoropa miliaris* tadpoles’ development using advanced techniques of microtomography and computational processing for segmentation of amphibian structures. From the radiographs acquisition, we obtained a 3D volume of the specimens in specific stages, and then a development study of the amphibians of these species was performed from the three-dimensional rendering of their internal morphology. The results are consistent with the expected morphology and size of developing organs, based on measurements of structures by dissection.

Our results have shown the value of phase-contrast CT and the possibility of its application in other biological studies. Its applicability may open avenues either for anurans applied environmental or health studies, as well for other vertebrates. The present study provides new insights and ways do handle quantitative ontogenetic studies, in addition to the use of such results in more classical ones, involving morphology and systematic. Among the health and environmental studies, we foresee the application of such studies in comparing the effects of substances in the ontogeny of the studied structures, via fine comparison of volumetric data. Therefore, the applicability of such a line of investigation may be broad in the near future.
